# Proteomics Analysis in Japanese Medaka *Oryzias latipes* Exposed to Humic Acid Revealed Suppression of Innate Immunity and Coagulation Proteins

**DOI:** 10.3390/biology11050683

**Published:** 2022-04-29

**Authors:** Victoria V. Yurchenko, Alexey A. Morozov, Bogdan A. Kiriukhin

**Affiliations:** 1 Laboratory of Physiology and Toxicology of Aquatic Animals, Papanin Institute for Biology of Inland Waters Russian Academy of Sciences, 152742 Borok, Russia; 2 AquaBioSafe Laboratory, University of Tyumen, 625003 Tyumen, Russia; bogdan.kirukhin67@gmail.com

**Keywords:** fish, protein profile, blood clotting, immunosuppression, stress response, humic substances, organic carbon

## Abstract

**Simple Summary:**

Humic acids are one of the main components of the natural organic matter in surface waters that give them brown color. These compounds are known to have positive effects on aquatic animals such as increased growth and stress resistance. At the same time, there is experimental evidence that humic acids, being natural xenobiotics, act as follows: they cause stress responses at the molecular level. Our aim was to study humic acid-related effects on fish by performing the proteomic analysis of the blood plasma from Japanese medaka exposed to humic acid in concentrations that can be found in natural waters. Results of the study showed that most of the plasma proteins in the exposed fish had a lower abundance compared to that of the intact fish; humic acid caused a reduction in circulating levels of complement components, coagulation factors, and their regulators.

**Abstract:**

Humic acids (HA), one of the major components of dissolved organic matter, can interfere with different metabolic pathways in aquatic animals, causing various biological effects. This study aimed to provide a molecular basis for HA-related responses in fish by analyzing changes in the blood plasma proteome following short-term exposure to environmentally relevant HA concentrations using the Japanese medaka *Oryzias latipes* Hd-rR strain as a model organism. Proteomics data were obtained by high-performance liquid chromatography with tandem mass spectrometry analysis employing a label-free quantification approach. HA caused dysregulation of proteins involved in various biological processes, including protein folding, signaling, transport, metabolism, regulation, immune response, and coagulation. The majority of the differentially abundant proteins were down-regulated, including those involved in humoral immunity and coagulation. HA caused the decrease of the complement cascade and membrane attack complex proteins abundance, as well as proteins participating in activation and regulation of secondary hemostasis. The most pronounced suppression was observed at the highest tested HA concentration.

## 1. Introduction

Humic acids (HA), one of the main natural organic matter fractions, contain various hydro- and lipophylic functional groups [1], making them reactive in the aquatic environment. In natural waters, humic substances’ concentrations, reported as total organic carbon, vary in a range from about 2 mg C/L in oligotrophic lakes [2] to 10–12 mg C/L in rivers and eutrophic lakes [2,3].

Various findings suggest that humic substances are an important environmental factor in freshwater ecosystems [4]. To date, a number of studies have been published describing different effects of humic substances in aquatic animals, both stress responses including expression of heat shock proteins, modulation of biotransformation enzymes, development of oxidative stress symptoms and hormone-like effects [5,6] and positive effects such as increased vigor, growth, lifespan, and stress resistance [7]. It is now apparent that humic substances can interfere with various metabolic pathways in freshwater organisms.

For data accumulation and understanding of the molecular mechanisms of fish responses, a proteomic approach can be beneficial; proteome changes may unravel more physiological responses to environmental challenges and help the search for diagnostic indicators of stressful conditions [8,9]. Mass-spectrometry-based protein profiling has become a potent tool in protein identification and quantification; the label-free quantification (LFQ) approach using the MaxLFQ algorithms can achieve the highest possible quantification accuracy regarding the relative amount of proteins in the samples [10].

This article describes a pilot study aimed to provide valuable knowledge of the changes in the fish blood plasma proteome in response to HA exposure. In this study, we analyzed the effects of environmentally relevant HA concentrations following short-term exposure using Japanese medaka *Oryzias latipes* (Temminck & Schlegel 1846) as a model organism. Its reference proteome is available [11], which was advantageous for the study.

## 2. Materials and Methods

### 2.1. Ethics Statement

This research complied with the Directive 2010/63/EU on the protection of animals used for scientific purposes and the Papanin Institute for Biology of Inland Waters ethical standards for the use of laboratory animals.

### 2.2. Fish Maintenance

Fertilized eggs of Hd-rR strain medaka were received through the Scientific Program of USGS Columbia Environmental Research Center and Papanin Institute for Biology of Inland Waters, and the breeding stock of these medaka has been maintained in our laboratory for five years. Adult medaka (40–45 weeks old) intended for this study were kept at 16:8 h photoperiod (light hours: 07:00 to 23:00 h) and 25 ± 1 °C in the semi-static conditions (changing half the volume daily) during one-month acclimation and the exposure period as described earlier [12].

### 2.3. Experimental Procedure

For 96 h the fish were exposed to Sigma-Aldrich humic acid 53680, CAS 1415-93-6, at 5, 40, and 80 mg/L nominal concentrations to cover the humic substances’ concentration range naturally occurring in aquatic ecosystems [13,14]. To control the amount of test compound dissolved in the water in each treatment, organic carbon concentrations were measured twice over the exposure period (before and after water renewal) according to the OECD recommendations [15], in duplicate, employing the potassium dichromate photometric method using a KFK-3 instrument (ZOMZ, Sergiyev Posad, Russia); dissolved oxygen and pH were measured daily in duplicate (Table 1).

Medaka were kept in 10 L chambers (one per treatment and control) at ≤0.5 g/L stocking density. Fish were fed TetraMin Mini Granules fish food four times a day using automatic feeders and *Artemia salina* nauplii twice a day manually.

### 2.4. Sampling

Eight individuals (4 males and 4 females) were used for blood plasma sampling in each treatment group and control. At the end of the exposure period, after anesthetization with tricaine methane-sulfonate (MS-222, 100 mg/L), each fish was weighed; mean values are given in Table 1. Blood was sampled from the caudal vein/artery. Each blood sample was collected into the centrifuge tube containing heparinized saline solution (concentration 5 mg/mL) and placed at 4 °C. Plasma was isolated by centrifugation for 10 min at 1500× *g* at 4 °C. Blood plasma aliquots from 8 fish from a treatment group were then pooled in one cryo-tube and frozen in liquid nitrogen for storage until the proteomic analysis could be performed.

Sample pooling obscures variation among individuals. The pooling process averages the distribution of proteins; certain proteins may be under the detection limit if one member of the pooling group does not express the proteins in question, leading to loss of information [16]. However, because the protein expression in a pool matches the mean expression in the individual samples making up the pool for most proteins, the pooling might help overcome resource constraints and be a valid and potentially valuable procedure in proteomics [17]. As pooling makes the main differences and similarities between groups easily detectable [18], we found this approach advantageous for the study.

### 2.5. High-Performance Liquid Chromatography with Tandem Mass Spectrometry (HPLC–MS/MS) Analysis

Proteomic analysis is described in detail in [19]. Briefly, total protein concentration in the plasma samples was measured employing the bicinchoninic acid assay. Trypsin protein digestion was carried out using S-Trap Mini Spin Columns (ProtiFi, Farmingdale, NY, USA) according to the manufacturer’s protocol. An aliquot of obtained peptides was loaded onto the Acclaim µ-Precolumn; then peptides were separated with HPLC UltiMate 3000 RSLCnano system (Thermo Fisher Scientific, Waltham, MA, USA) in the Acclaim PepMap RSLC column (Thermo Fisher Scientific, Waltham, MA, USA). The peptides were eluted (total run time 90 min). The MS analysis was performed using a Q Exactive HF-X Hybrid Quadrupole-Orbitrap mass spectrometer (Thermo Fisher Scientific, Waltham, MA, USA) (capillary temperature 240 °C, emitter voltage 2.1 kV). Mass spectra were acquired at a 120,000 resolution in a 300–1500 m/z range. Tandem mass spectra of fragments were acquired at a 15,000 resolution, from 100 m/z to the value determined by a charge state of a precursor (2000 m/z at the most). The maximum integration time values were 50 ms and 110 ms, and automatic gain control target values were 1 × 10^6^ and 2 × 10^5^, for precursor and fragment ions, correspondingly. An isolation intensity threshold of 50,000 counts was determined for precursor selection. Top-20 precursors were chosen for fragmentation with higher-energy collisional dissociation; normalized collision energy was set at 29. Precursors with a charge state of 1+ and more than 5+ were rejected, and all measured precursors were dynamically excluded from triggering a subsequent MS/MS for 20 s.

Each pooled blood plasma sample (=treatment) was analyzed in triplicate. Obtained data were processed using the MaxQuant software (version 1.6.3.4) with the built-in Andromeda peptide search engine. Protein sequences for *Oryzias latipes* provided by UniProt (February 2021) were used for protein identification. Peptide and protein identification false discovery rates were set at 5%. The relative amount of proteins in the samples, or protein abundance, was determined by the LFQ method.

### 2.6. Data Processing

Only proteins with minimally two peptides detected were considered reliably identified. Raw proteomic data were processed using Microsoft Excel, according to Aguilan et al. [20]. Briefly, LFQ intensities were log_2_-transformed to make the variances more constant and transform the data into a normal distribution. Then, to reduce intragroup variation in technical replicates and correct for artificial biases, the data were normalized in two steps, by median and by data distribution width, and the missing values were replaced with the probabilistic minimum imputation method (Supplementary Material—Table S1, data processing spreadsheet). Processed proteomic data were then used to calculate the ratio of the relative abundance of each protein between each treatment and control (fold change, FC). To assess the reproducibility of the results for each protein, first, an F-test was performed between each treatment and control (i.e., between three data points in a given treatment and three data points in control). Then, based on the obtained *p*-value, a *t*-test type 3 or type 2 was performed (Supplementary Material—Table S1, “data analysis” spreadsheet). These *t*-test *p*-values and a fold-change cutoff of 2 were applied to select differentially abundant proteins (DAPs). The results were visualized in volcano plots constructed in R (v4.1.2), where the statistical significance threshold of *p* < 0.05 was represented by −log_2_ (*p*) > 4.3219, and FC = 2 was represented by log_2_ (FC) = 1. The processed proteomics data were also used to construct heat maps in R (v4.1.2) with the ggplot2 package [21] and generate protein-protein association networks using the online STRING software [22]. Functional analysis of proteins was performed using the Proteus R package, “fetchFromUniProt” function [23], UniProtKB [24], PANTHER 16.0 [25], InterPro 87.0 [26], and STRING [22] databases. For fast comparison of protein sets between treatments, a Venny 2.1 analysis tool was used [27].

## 3. Results and Discussion

Organic carbon concentrations in the tested solutions were environmentally relevant; the highest tested concentration was equivalent to that found in eutrophic lakes and rivers [2,3].

According to the results of the HPLC–MS/MS analysis, a total of 164 proteins met the requirements for reliable identification in the pooled samples of medaka blood plasma (Supplementary Material—Table S1, “filtered by peptides” spreadsheet). From this set, 153 proteins quantified in at least 2 out of all 12 technical replicates were used for further analysis (Supplementary Material—Table S1, “data analysis” spreadsheet). The set of 153 proteins was uploaded to the STRING online software to generate a protein-protein association network. The resulting network comprised 136 nodes representing all the proteins produced by a single protein-coding gene locus listed in the database (Figure S1, Table S2—Supplementary Material). The network included 796 edges representing specific and meaningful protein associations, i.e., when proteins jointly contribute to a shared function and do not necessarily physically bind to each other [22]. Protein-protein interaction enrichment *p*-value was <1.0 × 10^−16^, which means that proteins have more interactions among themselves than expected for a random set of proteins of the same size and degree distribution drawn from the genome, confirming that the proteins are biologically connected as a group.

**Figure 1:**
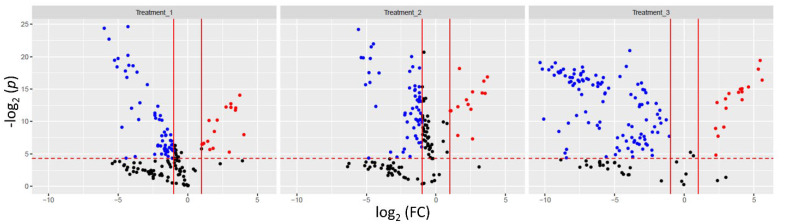
Volcano plots show a measure of statistical significance from a *t*-test and the magnitude changes of the relative abundance of proteins in the blood plasma of medaka exposed to HA; dots represent proteins: blue—decreased (FC < 0.5, i.e., log2 (FC) < −1, the *t*-test is significant), red—increased (FC > 2, i.e., log2 (FC) > 1, the *t*-test is significant), black—*t*-test is not significant or −1 < log2 (FC) < 1.

Proteomic changes between treatments are shown in Figure 1. Volcano plots represent DAPs in each treatment; the greatest number was observed in treatment 3, a total of 124 DAPs, while in treatments 1 and 2, the numbers were much smaller, 77 and 70, respectively (Supplementary Material—Table S1, “DAPs” spreadsheet).

Amongst the up-regulated proteins, 13 were shared by all three treatments: six apolipoproteins (H2MLX9, H2MFZ1, A0A3B3H5L1, H2MFX3, H2MG05 from the apolipoprotein A1/A4/E family, and A0A3B3IF59 from the apolipoprotein C1 family), three parvalbumins (H2M0U7, A0A3B3I979, and H2M0U0), ubiquitin (A0A3B3IL47), tropomyosin (A0A3B3I241), fetuin (H2LZ65), and an uncharacterized protein H2L6Q4, belonging to calycin superfamily. In treatment 1, additional four proteins were up-regulated: apolipoprotein M (A0A3B3HAM1) from the lipocalin family, an uncharacterized glycoprotein H2MUY7 belonging to the LEG1 family, superoxide dismutase [Cu-Zn] (H2MYT0), and an uncharacterized protein A0A3B3IEL6 presumably involved in the nuclear division [28]. The latter two proteins were up-regulated in treatment 3 also, as well as globin domain-containing protein (A0A3B3I6M9) and parvalbumin (H2LE63). The latter was also up-regulated in treatment 2.

Apolipoproteins’ molecular function is fatty acid and lipid binding; H2MLX9 is also known to increase the activity of phosphatidylcholine-sterol O-acyltransferase, and A0A3B3IF59 to have phospholipase inhibitor activity. Apolipoproteins are secreted plasma proteins involved in various processes related to lipid transport, metabolism, homeostasis, lipoprotein metabolism, and regulation of molecular functions [28]. Parvalbumins are intracellular Ca^2+^-binding proteins. They are not considered a classical component of fish blood plasma, but as “transit” proteins [29]. Interestingly, parvalbumin was identified in the seminal plasma of common carp *Cyprinus carpio*, but not in blood plasma, and is believed to be involved in the mechanism controlling carp sperm movement [30]. It was also identified in the frog *Rana temporaria* skin secretome and considered to be involved in prey recognition by snakes [31]. The plasma proteome comprises various functional groups of proteins, including tissue leakage products, proteins normally functioning within cells and released into plasma due to cell death or damage [32]. Since parvalbumin was amongst the DAPs in all treatments, it is a question for further research as to whether it could be a marker of HA-induced stress in fish. Ubiquitin and tropomyosin are also intracellular proteins; ubiquitin takes part in protein post-translational modifications, and tropomyosin is involved in actin filament organization and cardiac muscle contraction [33]. Fetuin is a component of the extracellular space and an endo-peptidase inhibitor [28]; it has been shown to function in many physiological aspects, including fatty acid transport, regulation of insulin activity, and response to systemic inflammation [34]. Extracellular superoxide dismutase (SOD3), a copper, zinc-containing enzyme in blood plasma and extracellular fluids, catalyzes superoxide radicals removal [35]. SOD3 up-regulation indicates activation of the defense mechanism against reactive oxygen species (superoxide radicals in particular) in the blood plasma of medaka. It has been established that HA is a potent oxidant, and reactive oxygen species generation is one of the proposed mechanisms of HA-induced oxidative stress [36]. Induction of oxidative stress defense proteins has been reported in fish subjected to the HA-related challenge. For instance, SOD activity was induced in gills of brown trout exposed to 5 mg/L HA for seven days [37], and dietary humic substances administered for nine weeks increased mid-gut antioxidant enzymes activities, including SOD, in Nile tilapia [38]. Our results also agree with the paradigm of pro-oxidant action of HA within a biological system. Globin domain-containing protein (A0A3B3I6M9), part of a hemoglobin complex, is also a leakage product normally restricted to erythrocytes [39].

Most DAPs were down-regulated (Figure 1); 33 proteins were shared by all treatments: eight complement components—C4B (H2LRL9 and H2LRP2), C3 (H2M6U1, H2M6Q1, and H2N0N0), C5 (H2MT65), C6 (H2MIX9), and a protein belonging to the C6/C7/C8/C9 family (A0A3B3HA48), three regulatory proteins of complement or coagulation activation—factor B (P79816), factor P (H2LMQ3), and serpin (H2LN13), two coagulation factors—factor VII (H2L6K2) and fibrinogen γ-chain (H2LW76), olfactomedin 4 (A0A3B3I5S3), a glycoprotein regulating a variety of cell signaling pathways and essential biological functions [40], alpha-2-macroglobulin-like protein (H2M2A3) and alpha-1-antitrypsin homolog (H2MMF6) exhibiting serine-type endo-peptidase inhibitor activity, hepatocyte growth factor-like protein (H2LF02) involved in the regulation of receptor signaling pathway via JAK-STAT, hemopexin (H2LFK2), a protein that binds heme and transports it to the liver for breakdown and iron recovery, lecithin-cholesterol acyltransferase (H2MAY7) involved in the lipid metabolic process, sulfhydryl oxidase (H2MSG5) taking part in protein folding, fetuin-B (H2MAG7), whose molecular function is cysteine-type endo-peptidase inhibitor activity, apolipoprotein B-100 (A0A3B3IC86) involved in lipoprotein transport and cholesterol homeostasis, vitelline membrane outer layer protein 1 (A0A3B3HTH5), a female plasma protein domain [41], four uncharacterized proteins (A0A3B3I0Q1, A0A3B3HNN4, H2L522, and H2LER6), and 6 intracellular proteins with various functions [28]. The group of intracellular proteins included fructose-bisphosphate aldolase (H2LPL6), a cytosol protein of the glycolytic process, cytosolic non-specific dipeptidase (H2LF82), participating in proteolysis, heme-binding protein 3 (L0N757), also referred to as heme-binding protein 2-like, a protein with uncovered functions, belonging to the “SOUL family of proteins related to cellular fate” [42], L-lactate dehydrogenase (A0A3B3I1R7), an oxidoreductase known to take part in the anaerobic metabolic process by catalyzing the reversible conversion of lactate to pyruvate [43], uridylate-specific endoribonuclease (H2M5U1) that catalyzes the hydrolysis of ester linkages within RNA [28], and choriogenin H minor (H2M2K4) produced in the female liver and converted to zona pellucida-2 protein during oogenesis in medaka ovaries [44]. Interestingly, HA exposure caused down-regulation of vitelline membrane outer layer protein 1, a basic protein present in the outer layer of the vitelline membrane of eggs forming a barrier of fibrous layers and preventing infection from bacteria [41], and choriogenin H minor, a protein crucial for the normal oogenesis and spawning, as it takes part in the construction of the oocyte’s fibrous membrane structure [44]. Thus, the impact of HA on biological processes supporting fish reproduction seems a promising direction for further research.

**Figure 2:**
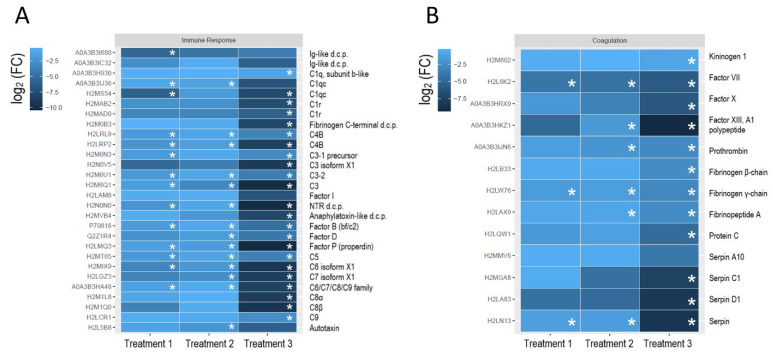
Heat maps show the relative abundance of proteins involved in the immune response (**A**) and coagulation (**B**). Each colored box represents the relative abundance of a protein according to the color key. Differentially abundant proteins (DAPs) are indicated with asterisks. IDs column provides protein identifiers (accession numbers of the UniProtKB entries). If an identified protein had no assigned protein or gene name in the UniProt or PANTHER databases (uncharacterized protein), we provided a protein family/subfamily name; d.c.p.—domain-containing protein.

Functional analysis of all 153 identified proteins showed that HA exposure caused the down-regulation of proteins involved in the immune response and coagulation (Figure 2). Amongst 28 identified proteins supporting immune functions (Figure 2A), 27 were constituents of the complement system, and one was an uncharacterized protein H2L5B8 belonging to the ectonucleotide pyro-phosphatase/phosphodiesterase family member 2 subfamily [33], also known as autotaxin. Blood plasma samples from treatments 1 and 2 had 14 DAPs each, and 23 DAPs were recorded in treatment 3. The most pronounced response to HA exposure in treatment 3 was evident not only in the count of DAPs but also in the magnitude of down-regulation. While changes in the relative abundance of autotaxin, immunoglobulin (Ig)-like domain-containing proteins, factor I, and one out of two identified C1qc subcomponents were not significant (*t*-test, *p* > 0.05), the magnitude of dysregulation (log2 (FC)) amongst DAPs in treatment 3 varied from −1.3, for C1q subcomponent subunit b-like, to −10.4, for factor P (properdin).

Autotaxin is a secreted glycoprotein that catalyzes the hydrolysis of lysophosphatidylcholine to lysophosphatidic acid, which participates in many processes, including lymphocyte trafficking and immune regulation [45]. Identified Ig-like domain-containing proteins, parts of the circulating immunoglobulin complexes, are responsible for antigen and Ig receptor binding and participate in several biological processes, including complement activation, classical pathway, and positive regulation of B cell activation [28]. The complement system, one of the humoral components of innate immunity, is a cascade of circulating proteins that act cooperatively to mediate defense mechanisms, including eliminating pathogens through opsonization and phagocytosis and promoting the inflammatory response [46]. The overwhelming majority of the identified complement components was significantly suppressed (*p* < 0.05) at the highest tested HA concentration.

C1q and C1r are components of the C1-complex whose activation initiates the classical complement pathway [47]. Fibrinogen C-terminal domain-containing protein enables antigen binding and is involved in the lectin complement pathway [28]. C4B, C3, and C5 components, NTR and anaphylatoxin-like domain-containing proteins, factors I, B (bf/c2), D, and P are involved in the activation of the further steps of the complement cascade [48]. Complement components C6, C7, C8α, C8β, and C9 are the terminal pathway proteins, components of the membrane attack complex [49]. Each complement pathway (classical, alternative, and lectin) comes to a point of cleavage of the C3 component, followed by the formation of a C5 convertase, which initiates the formation of the lytic membrane attack complex that destroys or damages targeted cells [50]. In fish, innate immunity is a fundamental defense mechanism [51]. Therefore, the changes in one of the innate immune system key components may severely affect fish health.

The complement system regulation is performed by several circulating and membrane-bound complement inhibitors. It is well established that proteins that either share structural homology to complement inhibitors or extracellular matrix macromolecules that interact with and modulate complement activity can be responsible for the complement system regulation [52]. Therefore, it is possible that some structural components of complex HA compounds can mimic these molecules and thus interfere with complement activation causing complement deficiency. Another possible explanation for the reduction in circulating levels of complement components in fish might be the HA ability to modulate bacterial community composition in the fish skin mucus and potentially protect them against pathogen invasion [53]. It had been shown earlier in studies on aquatic vertebrates that, in agreement with the diversity-invasibility hypothesis [54], hosts with a more diverse skin bacterial community have comparatively fewer cutaneous infections [55]. Therefore, the ability of HA to increase bacterial diversity of the skin mucus, a primary barrier against pathogenic invasion, can contribute to fish being less prone to invasion by pathogens [53]. However, this explanation of our results seems less probable considering the significant deficiency of complement components and regulating proteins at the highest tested HA concentration.

According to the functional analysis of proteins, 13 identified proteins are involved in coagulation (Figure 2B); these proteins were also down-regulated in response to HA exposure. Similar to the “immune response” subset, the most pronounced down-regulation was observed at the highest tested HA concentration; DAPs number increased from 3 to 6 to 12 in treatments 1–3, respectively. In treatment 3, statistically significant down-regulation magnitude varied from −1.1 (kininogen) to −9.3 (coagulation factor XIII).

In teleost fish, coagulation begins in a few seconds with thrombocyte plug formation; thrombocytes adhere to the collagen fibers of a blood vessel damaged site, and the plasma components respond in a complex cascade to form the fibrin reinforcing the thrombocyte plug [56]. As in mammals, three pathways are distinguished: intrinsic, extrinsic, and the final common pathway. Following the HA exposure, our results showed the most significant dysregulation magnitudes for factors XIII, X, and VII and three serpin domain-containing proteins in the blood plasma sample from treatment 3. Coagulation factor VII, a component of the extrinsic pathway, is responsible for starting a cascade of proteolytic events that lead to thrombin generation, fibrin deposition, and platelet activation [57]. Factor X is a common component of extrinsic and intrinsic pathways of the coagulation cascade. Activated factor X as part of the prothrombinase complex converts prothrombin to thrombin, which, in turn, activates factor XIII [58]. Identified serpin domain-containing proteins inhibit the activity of serine-type endo-peptidases [28] and thus regulate blood coagulation. Another down-regulated coagulation factor was protein C, a serine protease with potent anticoagulant, pro-fibrinolytic and anti-inflammatory properties, converted by thrombin to form activated protein C and acts by inhibiting activated factors V and VIII required for fibrin generation [56,58]. Amongst DAPs in treatment 3, kininogen 1, prothrombin, fibrinogen, and fibrinopeptide A showed smaller magnitudes of down-regulation. Kininogen, the precursor of inflammatory mediator bradykinin, is a component of the intrinsic pathway [56,59]. Prothrombin, fibrinogen, and fibrinopeptide A are proteins of the common pathway. Prothrombin is converted to thrombin, which cleaves circulating fibrinogen to its active form, fibrin; conversion of fibrinogen into fibrin occurs in four stages, at one of which fibrinopeptide A is released [60].

Descendants of a common ancestral pathway, complement and coagulation systems are composed of serine proteases with common structural characteristics; both systems belong to a complex inflammatory network and exhibit some similarities regarding specialized functions of their activators and inhibitors [61]. Many proteins involved in the innate immune response interact with or are controlled by the coagulation system [62]. It has been established, for instance, that the complement and coagulation cascades are intertwined in the extrinsic protease pathway, where some activated coagulation proteins, including thrombin, can directly cleave complement components C3 and C5, bypassing the canonical activation pathways [49].

Although no clear concentration-dependent pattern in the proteins’ dysregulation was observed, most proteins supporting immune and coagulation pathways were severely down-regulated at the highest tested HA concentration. Thus, it seems evident that HA can affect the humoral defense and coagulation system compromising fish health. Unless a fish had an effective hemostatic mechanism, when injured, it would suffer from blood loss, meaning excessive energetic demands which could undermine other physiological processes. Furthermore, with the suppressed complement system, fish’s defense against pathogenic agents would be considerably weakened. Immunosuppression is one of the tertiary stress responses, whose development, if the stressor persists over time, can lead to the depletion of energy reserves and defense systems, behavior and reproduction impairments [63].

## 4. Conclusions

Taken together, the results of this preliminary study of proteomic changes in the blood plasma of medaka exposed to environmentally realistic HA concentrations suggest that this natural xenobiotic ubiquitous in surface waters can dysregulate proteins involved in various biological processes, including protein folding, signaling, transport, metabolism, regulation, immune response, and coagulation. Proteins belonging to the complement and coagulation pathways showed unanimous down-regulation, with various degrees of reproducibility of the results, in response to the HA challenge. Therefore, further studies of short- and long-term HA effects on the immunity and other aspects of fish physiology are important for a better understanding of environmental limitations for fish welfare.

## Figures and Tables

**Table 1:** Mean values of monitored parameters of tested solutions and fish weight.

Treatment	HA, mg/L, Nominal	Organic Carbon, mg C/L, 0 h	Organic Carbon, mg C/L, 78 h	pH (±SD)	O_2_, mg/L, (±SD)	*W*, g, (±SD)
Control	0	<LOQ	<LOQ	8.14 ± 0.16	6.6 ± 0.3	0.49 ± 0.08
1	5	<LOQ	<LOQ	7.98 ± 0.43	6.6 ± 0.3	0.50 ± 0.07
2	40	4.5	6.6	8.10 ± 0.24	6.6 ± 0.2	0.50 ± 0.11
3	80	9.4	9.4	8.16 ± 0.22	6.7 ± 0.2	0.50 ± 0.05

*W*—total wet weight; LOQ—limit of quantification.

## Data Availability

Raw mass-spectrometry data were deposited to the ProteomeXchange Consortium via the jPOST partner repository [64]; the dataset identifiers are PXD025857 for ProteomeXchange and JPST001159 for jPOST (accessed date 12 March 2022). MaxQuant output and processed proteomics data are available in the Supplementary Material section (Table S1).

## Supplementary Materials

The following supporting information can be downloaded at: https://www.mdpi.com/article/10.3390/biology11050683/s1, Figure S1: STRING protein-protein association network generated using proteins identified in the blood plasma of medaka exposed to humic acid; Table S1: Microsoft Excel spreadsheets containing processed proteomics data; Table S2: Microsoft Excel spreadsheets containing STRING network legend.
