# Supplementary figures and images for: Proteomics Analysis in Japanese Medaka Oryzias latipes Exposed to Humic Acid Revealed Suppression of Innate Immunity and Coagulation Proteins
Source: Biology (Basel). 2022 Apr 29;11(5):683. doi: 10.3390/biology11050683 (PMC9138666; doi:10.3390/biology11050683)

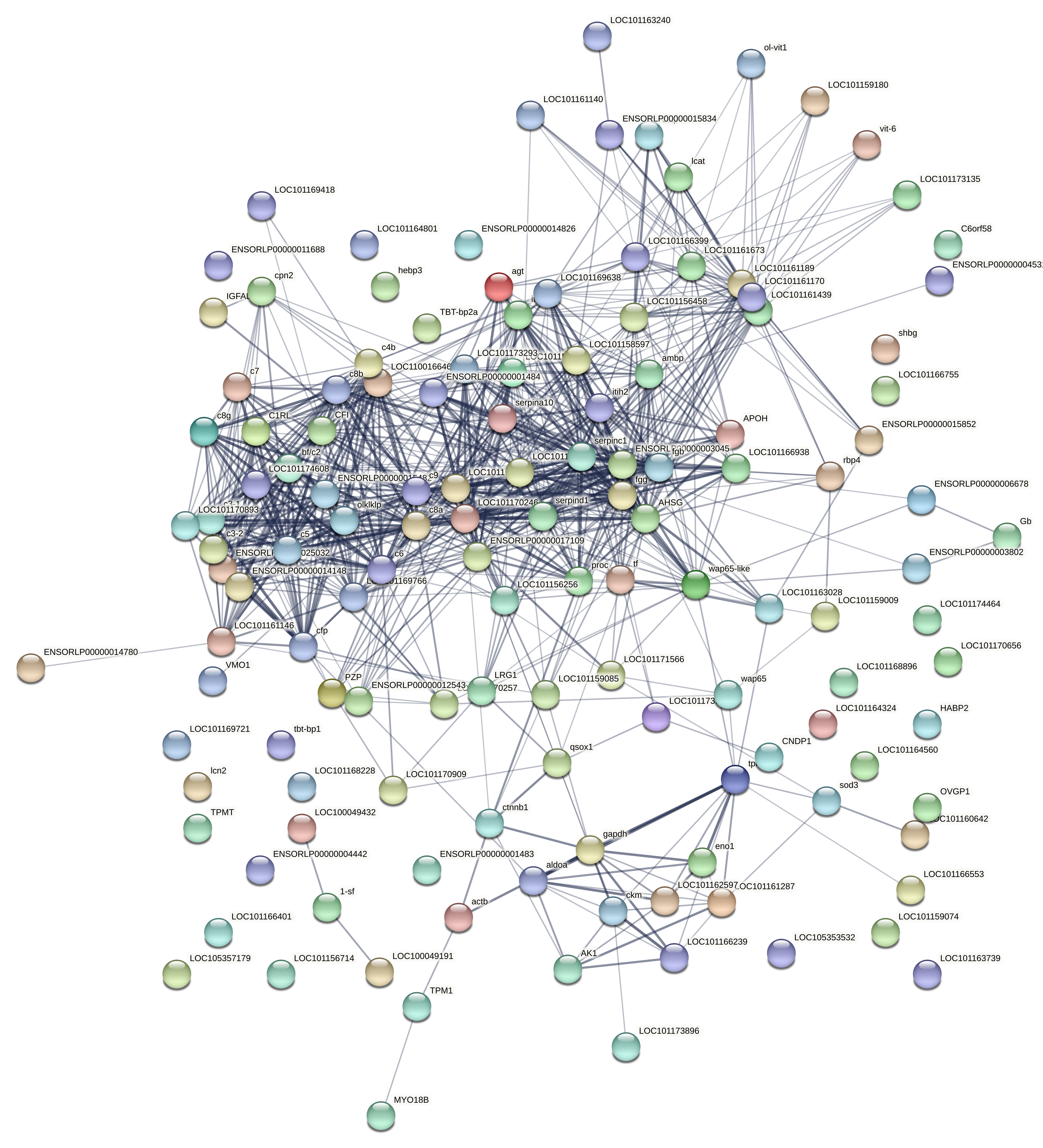

Supplement: Supplementary file 1 [file biology-11-00683-s001.zip › Supplementary data/Figure S1_STRING network.png]
